# Functional redundancy, complementation, and cross-talk between CH25h and IFITM3 in infection and in IFITM3 trafficking and secretion

**DOI:** 10.1128/jvi.01378-26

**Published:** 2026-09-28

**Authors:** Yuxin Song, Tingting Xia, Charlène Martin, Gaetan Despres, Julien Burlaud-Gaillard, Antonin Lamaziere, Philippe Roingeard, Andrea Cimarelli

**Affiliations:** 1 Centre International de Recherche en Infectiologie (CIRI), Université de Lyon, Inserm, U1111, Université Claude Bernard Lyon 1, CNRS, UMR5308, École Nationale Supérieure de Lyon, Lyon, France; 2 Saint Antoine Research Center, Sorbonne Université, INSERM UMR 938 27063, Paris, France; 3 Plateforme IBiSA de Microscopie Electronique, Université de Tours et CHU de Tours, Tours, France; 4 INSERM U1259, Université de Tours et CHU de Tours, Tours, France; 5 Département de Métabolomique Clinique, Hôpital Saint Antoine, AP-HP, Sorbonne Université, Paris, France; The Ohio State University, Columbus, Ohio, USA

**Keywords:** cholesterol, IFITM3, HIV, VSV, DENV, WNV, virus, membrane, inhibition

## Abstract

Type I interferon induces multiple protein effectors to inhibit viral replication. Among these, the interferon-induced transmembrane protein (IFITM3) and the cholesterol-25-hydroxylase (CH25h) converge on the inhibition of viral entry by altering the behavior of membranes. Here, we dissect the functional and mechanistic relationship between these two membrane-acting effectors using different viruses. When using HIV-1 and vesicular stomatitis virus (VSV), IFITM3 and CH25h restrict viral entry with similar efficiency but act in a largely redundant manner, a finding that is consistent across infection systems, cell types, and entry assays. However, this is not the case for several orthoflaviviruses against which IFITM3, but not CH25h, provides a strong block to infection. We further uncover that the CH25h-25HC axis remodels IFITM3 trafficking by driving partial relocalization of IFITM3 from endolysosomal compartments to the plasma membrane. This occurs by impairing IFITM3 internalization into early endosomes and leads to an increase in its secretion in exosomal vesicles, overall indicating that CH25h contributes to regulate IFITM3 cellular dynamics. Together, these findings reveal that the apparent redundancy of maintaining two distinct effectors targeting the same step of the viral life cycle is not superfluous but instead allows the cell to compensate and, nonetheless, target viruses that may have developed resistance to one of these effectors. Furthermore, the ability of CH25h to remodel IFITM3 trafficking indicates that these two pathways are connected, adding an additional layer of complexity in the biology of IFITM3.

**IMPORTANCE:**

IFITM3 and CH25h are two potent, broad-acting inhibitors of viral membrane fusion, but their functional connections have not been explored. Here, we show that although their antiviral activities are largely redundant against HIV-1 and vesicular stomatitis virus (VSV), IFITM3 functionally compensates for a relative resistance of orthoflaviviruses toward CH25h. Furthermore, CH25h redistributes IFITM3 from endosomal compartments to the plasma membrane, enhancing its release in exosomal vesicles. Overall, these findings reveal that despite the fact that IFITM3 and CH25h can act redundantly against certain classes of viruses, IFITM3 can functionally compensate for CH25h resistance against other viruses, highlighting why it is important for the cell to maintain two distinct pathways to block virus-to-cell membrane fusion. In addition, these results also reveal how interferon-driven lipid remodeling can reshape the trafficking behavior of IFITM3, leading it to be more present in exosomes, where it could exert paracrine functions that remain to be explored.

## INTRODUCTION

Type I interferon (IFN-I) responses constitute the first line of defense that cells deploy against viral infection. Once triggered, hundreds of IFN-induced gene products then interfere either directly, or indirectly, with viral replication. Among these, two antiviral effectors stand out for their capacity to restrict viral entry by altering the properties of membranes: interferon-induced transmembrane proteins (IFITMs) and cholesterol-25-hydroxylase (CH25h).

IFITM1, IFITM2, and IFITM3 are the most prominent antiviral members of the IFITM family. IFITMs are membrane-bound proteins and share a typical domain organization with one intra- and one transmembrane domain separated by an intracellular loop (IMD, TMD, and CIL, respectively). They also possess N and C-termini of variable lengths that tightly regulate IFITM trafficking, stability, and post-translational modifications (1–3).

Functionally, IFITMs inhibit viral replication at two distinct stages. In target cells, endosome-resident IFITMs block low-pH-dependent fusion between viral and endosomal membranes, leading to virion degradation (4–8). In virus-producing cells, IFITM expression results in the release of virions that are intrinsically less fusogenic and, therefore, less infectious (9–11). Although the precise mechanism underlying this fusion defect remains incompletely understood, current evidence suggests a combination of direct membrane-rigidifying effects driven by IFITM insertion and multimerization, coupled with possible indirect effects on membrane composition (5–7, 12–14).

CH25h represents another IFN-induced antiviral defense acting at membranes. This endoplasmic reticulum (ER)-resident enzyme catalyzes the conversion of cholesterol into 25-hydroxycholesterol (25HC), a soluble oxysterol with paracrine and autocrine immunomodulatory functions (15–17). 25HC is a potent broad-spectrum antiviral factor and, similar to IFITMs, inhibits viral entry by impairing virus-to-cell membrane fusion—an effect thought to arise from the incorporation of its bulky, hydroxylated head group into cellular membranes (16, 18, 19).

Because IFITMs and CH25h inhibit a wide range of viruses at the same early fusion step, we sought to determine whether they act redundantly, synergistically, or influence each other’s cellular behavior.

The results we have gathered indicate that IFITM3 and CH25h exert largely redundant effects on the replication of representative members of the *Retroviridae* and *Rhabdoviridae* (HIV-1 and vesicular stomatitis virus [VSV]), under the conditions used here. This does not appear to be the same for several orthoflaviviruses tested here that instead resist CH25h expression but remain susceptible to IFITM3.

Furthermore, we identify a previously unrecognized connection between the two pathways: the CH25h-25HC axis substantially redistributes IFITM3 from internal endosomal compartments to the plasma membrane. This relocalization correlates with a general block of dynamin-dependent endocytosis and results in an increased secretion of IFITM3 in exosomal vesicles. These findings reveal a novel mode of cross-regulation in which CH25h influences the trafficking and extracellular release of IFITM3, independently of their direct antiviral activities.

Overall, our study demonstrates that although IFITM3 and CH25h do not act additively to restrict HIV-1 or VSV infection, they influence each other’s biological properties in ways that may have broader implications for membrane biology, innate immunity, and vesicle trafficking.

## RESULTS

### CH25h and IFITM3 restrict HIV-1 in a largely non-cooperative manner

To define how CH25h and IFITM3 functionally relate during the HIV-1 life cycle, we first examined viral spread using replication-competent NL4-3 virus in SupT1 cells expressing doxycycline-inducible IFITM3 and treated with concentrations of 25HC ranging from 0.17 to 1.5 μM (higher concentrations were avoided following pilot experiments pointing to toxicity in this cell type).

25HC treatment did not affect the steady-state accumulation of IFITM3, as assessed by Western blot (WB), nor did it alter cell proliferation in the timeframe of our spreading infection assays (Fig. A1A and B).

**Fig 1:**
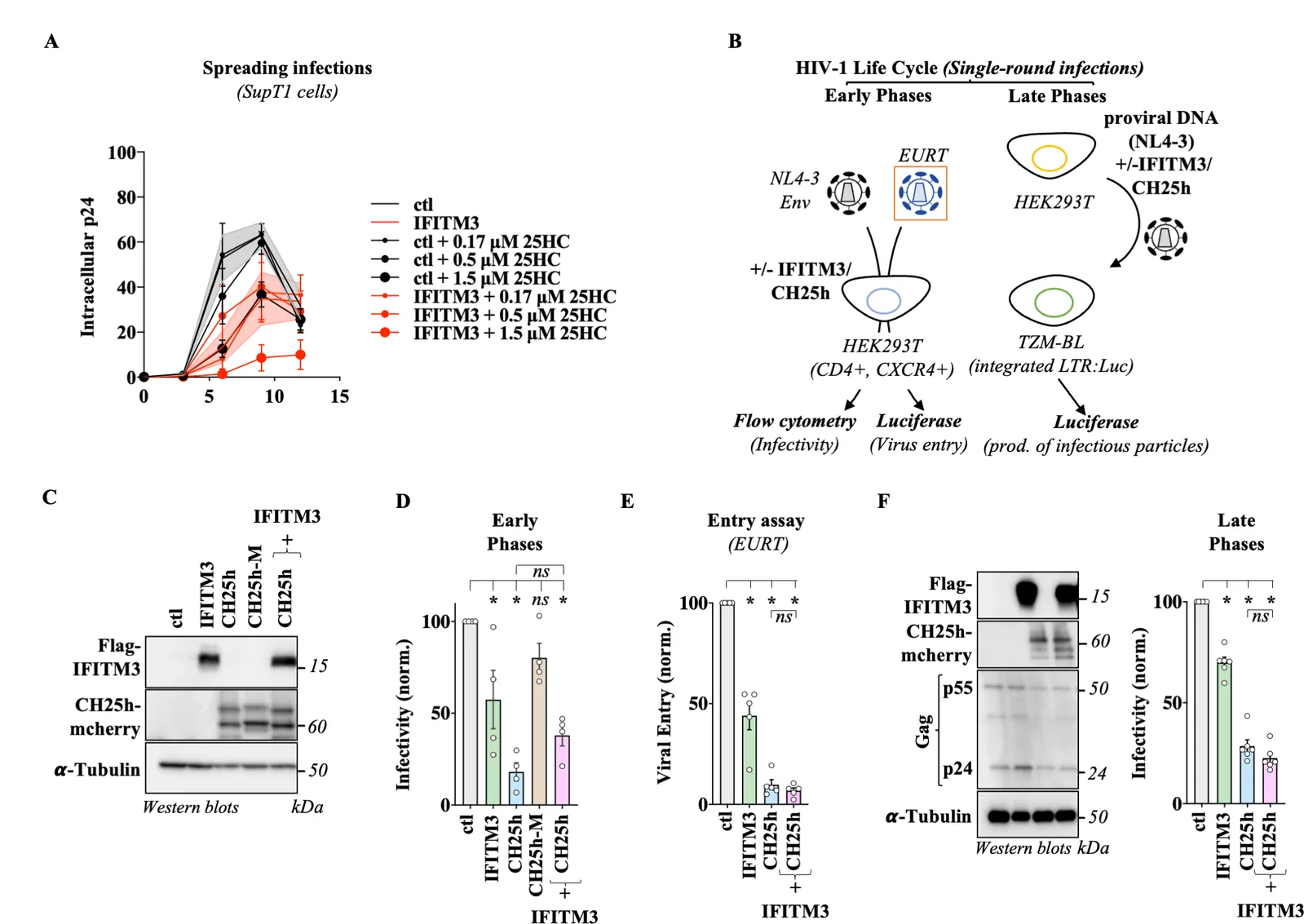
IFITM3 and CH25h exert largely redundant roles during HIV-1 infection. (**A**) Control or dox-inducible IFITM3 stable SupT1 cells were treated with doxycycline (1 μg/mL), +/− 25 HC prior to viral challenge with an MOI of 0.1. Cell aliquots were harvested at the indicated time points, and the percentage of infected cells was measured by FACS following intracellular p24 staining. (**B**) Experimental scheme used here to examine the role of IFITM3 and CH25h on the early and late phases of HIV infection. In the former, HEK293T expressing the CD4/CXCR4 HIV-1 receptor/co-receptors were first transfected with plasmids coding the indicated combinations of Flag-tagged IFITM3, wild-type CH25h-mCherry, or its catalytically inactive mutant CH25h-M-mCherry. After 36 h, cells expressing the different proteins were either analyzed by WB (**C**) or challenged with a single round of infection-competent NL4-3 Env-pseudotyped HIV-1 virus coding a CMV:GFP reporter cassette. The extent of infection was determined 2 to 3 days later by flow cytometry (**D**). To more specifically measure defects in virus entry, viruses incorporating a viral genome mimic coding for the Firefly Luciferase that can be directly translated upon entry of the virus into the cell were used to challenge target cells for 18 h in the presence of the capsid destabilizing agent PF74 (10 μg/mL). The extent of virus entry into the cells was measured in function of the accumulation of Luciferase activity in cell lysates (**E**). The late phases of HIV-1 infection were studied by directly producing HIV-1 viruses in HEK293T cells by coexpression of the DNAs indicated above together with the proviral DNA construct NL4-3, which allows the production of fully replicative virus. Infectious virus was harvested from these producing cells, diluted 100 times to nullify the effects of 25HC on the new cycle of infection, and used to challenge TZM-BL target cells. The extent of production of infectious virus was determined after measurement of the luciferase activity thanks to the LTR:Luciferase reporter integrated in TZM-BL cells (**F**). The WB panels depict typical results obtained, while graphs present average (AVG) and standard error of the mean (SEM) from independent experiments (C, *n* = 4; D, *n* = 4; E, *n* = 5; F, *n* = 6). * and ns, *P* < 0.05 or nonsignificant following ordinary one-way ANOVA and Dunnett’s multiple comparison tests.

In this long-term replication setting, both IFITM3 expression and 25HC treatment reduced HIV-1 spread, and in the case of 25HC, inhibition was proportional to its concentration. When combined, 25HC treatment of IFITM3-positive cells resulted in a stronger inhibition than either alone, but this occurred only at the highest concentration of 25HC used (Fig. 1A). This effect could be due to either a true synergistic effect between IFITM3 and 25HC or else due to pleiotropic effects of 25HC. To discriminate among these possibilities, we measured their effects on the early and late stages of infection using single-cycle assays, according to the scheme of Fig. 1B. To examine the early phases of the viral life cycle, HEK293T-CD4-CXCR4 cells expressing IFITM3 and/or a wild-type mCherry-CH25h protein alone or in combination were challenged with a single round of infection-competent HIV-1 viruses pseudotyped with the HIV-1 envelope protein derived from the NL4-3 strain and coding for a green fluorescent protein (GFP) reporter gene. A catalytically inactive CH25h mutant (CH25h-M, containing the following mutations: H242E/H243E/H246E/H247E) was included as the control (20). At 2 to 3 days post-infection, cells were either analyzed by WB (Fig. 1C) or by flow cytometry to determine the extent of infection (Fig. 1D). Under these conditions, each protein was robustly expressed, and no detectable changes were observed when the two proteins were expressed together (Fig. 1C). As expected, the expression of either IFITM3 or CH25h significantly inhibited the early steps of HIV-1 infection (Fig. 1D). CH25h expression led to higher inhibition of HIV than IFITM3 (18.0% versus 57.3%, respectively, Fig. 1D for normalized values and Fig. A1C for non-normalized ones), while the catalytically inactive CH25h-M mutant displayed no significant antiviral activity. Co-expression of IFITM3 and CH25h did not further enhance inhibition beyond the activity of CH25h alone (37.9%), and if anything, it was lower when compared to that of CH25h alone (18.0%), albeit this difference did not reach statistical significance.

To confirm that viral inhibition mapped at the step of HIV-1 entry into the cell, we used the entry/uncoating EURT assay, in which the translation of a virion-incorporated luciferase minigenome serves as a direct marker of virus entry into the cell (21). Cells are challenged with this virus, and capsids are destabilized by incubating target cells with PF74, prior to luciferase quantification 18 h later. Under these conditions, we found that IFITM3 and CH25h reduced early HIV-1 entry (Fig. 1E), in line with data present in the literature (4–8, 16). However, no cumulative effects were observed upon their co-expression.

Next, we assessed the effects on late stages of replication by producing virions from cells expressing IFITM3 and/or CH25h and measuring infectivity of newly produced virion particles on TZM-BL reporter cells (scheme of Fig. 1B and F). After 48 h, the virus was harvested, diluted 100 times, and then used to challenge TZM-BL target cells that bear an integrated LTR:luciferase reporter. The dilution step was introduced following pilot experiments to discard the effects of soluble 25HC present in the supernatant of virus-producing cells that could have influenced the interpretation of the results by influencing virus entry in target cells, rather than the infectivity of newly produced virions. Virus infectivity was measured 24 h after viral challenge of TZM-BL cells by quantifying the amount of luciferase produced, which in these cells is directly linked to the production of Tat from newly integrated proviruses. Under these conditions, virions produced in the presence of IFITM3 or CH25h exhibited reduced infectivity (66.0% and 24.2% of control, respectively). Again, co-expression resulted in inhibition comparable to that of CH25h alone (18.9%), indicating limited cooperation at this stage.

Together, these findings reveal, first, an undescribed effect of the CH25h-25HC axis on the late phases of HIV-1. Second, they demonstrate that IFITM3 and CH25h restrict HIV-1 through overlapping mechanisms, with negligible additive effects during single-cycle infection. Therefore, the additive effects observed during spreading infections are likely a consequence of the pleiotropic effects that 25HC may have on cell physiology upon prolonged culture.

### CH25h and IFITM3 inhibit VSV infection in a similarly redundant fashion

**Fig 2:**
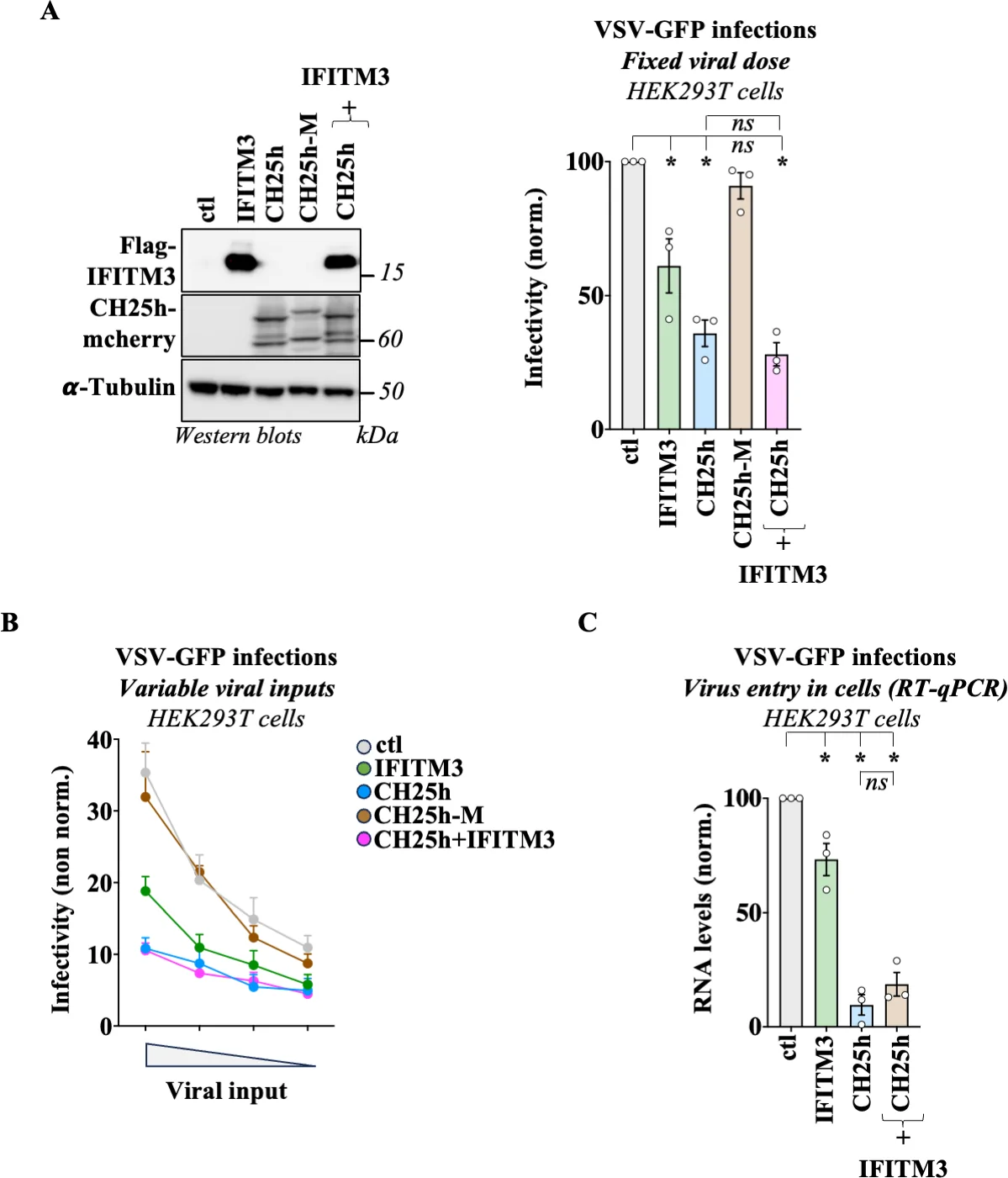
IFITM3 and CH25h exert largely redundant roles also against VSV. (**A**) HEK293T cells transfected with the indicated plasmids were either collected for WB analysis or challenged with VSV-GFP at an MOI of 0.05 for 16 h, followed by flow cytometry analysis. (**B**) A similar setup was employed using dilutions of the viral input. (**C**) The extent of virus entry into the cell was determined by measuring the levels of VSV-GFP RNA in cells 20 min after viral challenge. Cells were trypsinized and washed to remove unbound virus. The WB panels present typical results obtained. The graphs present AVG and SEM from independent experiments (A and C, *n* = 3; B, *n* = 6). * and ns, *P* < 0.05 or nonsignificant, respectively, following ordinary one-way ANOVA and Dunnett’s multiple comparisons tests.

Because IFITM3 and CH25h target membrane fusion, we assessed whether their relationship was conserved for viruses with different entry and replication strategies. To this end, we used a replicative VSV (Indiana serotype) bearing a GFP reporter transcription unit inserted between M and G (22). HEK293T cells expressing IFITM3 and CH25h (WT or M), alone or in combination, were challenged with a fixed amount of VSV, and the extent of infection was then measured 24 h later by flow cytometry (Fig. 2A for WB and normalized infection data and Fig. A2A for non-normalized infectivity data). As observed for HIV-1, both IFITM3 and CH25h markedly decreased susceptibility to VSV (Fig. 2A, 61.1% and 35.9% of control, respectively), whereas mutations altering the enzymatic activities of CH25h led to a loss of antiviral activity (90.99% of control). Co-expression of IFITM3 and CH25h inhibited infection but did not further increase restriction compared with CH25H alone (28.07%). These effects were maintained across different viral inputs (Fig. 2B) and were not cell type-dependent as similar effects were also obtained in A549 cells (Fig. A2B).

To map the inhibition step, we quantified incoming viral RNA 20 min post-infection in the presence of cycloheximide to block secondary RNA synthesis (Fig. 2C). Both IFITM3 and CH25h reduced the accumulation of incoming nucleocapsids, confirming a block at viral entry. Together, these results show that IFITM3 and CH25h restrict VSV entry in a largely non-additive manner, consistent with a shared mechanism of fusion inhibition.

### IFITM3, but not CH25h, inhibits the replication of several orthoflaviviruses

**Fig 3:**
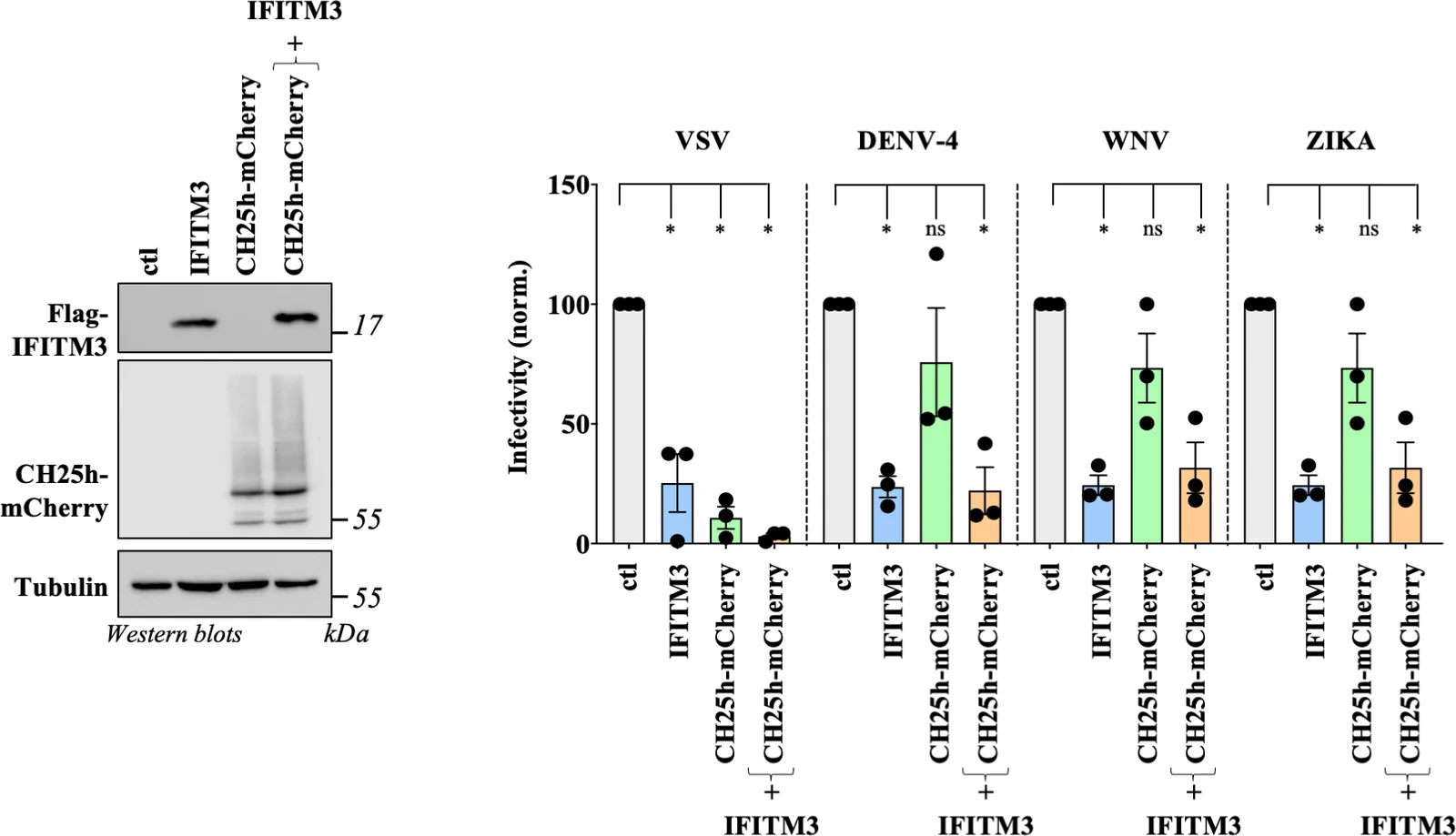
Several orthoflaviviruses exhibit relative resistance to CH25h but are susceptible to IFITM3-mediated inhibition. A549 cells expressing CH25h, IFITM3, or both were challenged with the indicated viruses with MOIs of 0.1 and 0.5 (for WNV and the other viruses, respectively). The percentage of infected cells was determined following intracellular staining with a pan-flavivirus Env antibody and flow cytometry analysis 24 h post-viral challenge. The WB panels present the pattern of expression of the indicated proteins, while the graph presents infection results obtained in three independent experiments. * and ns, *P* < 0.05 or nonsignificant, respectively, following ordinary one-way ANOVA and Dunnett’s multiple comparisons tests.

To determine whether a similar redundant behavior could be observed on more divergent viruses, we examined the susceptibility of the dengue virus (DENV), the West Nile virus (WNV), and the Zika virus to IFITM3 and CH25h alone or in combination in A549 cells in a side-by-side comparison with VSV. These viruses are positive-strand RNA viruses that belong to the *Orthoflavivirus* genus and were chosen because they assemble in the ER-Golgi, compartments with different lipid composition than the plasma membrane. Surprisingly, we observed that while all were susceptible to IFITM3, in line with previous studies (4), the three orthoflaviviruses tested exhibited little to no inhibition toward CH25h, in contrast to the strong inhibition observed for VSV under identical experimental conditions (Fig. 3). While this is in apparent contrast with a previous study that pointed to the CH25h/25HC axis as inhibitory for these viruses (18), our results, nonetheless, indicate a differential susceptibility to CH25h between members of various viral families and indicate that orthoflaviviruses are largely resistant to it. Overall, these results also suggest a potential advantage for the cell in maintaining redundant mechanisms of inhibition against viruses that exhibit resistance against one of them.

### IFITM3 does not modulate the secretion of 25-hydroxycholesterol

To determine whether IFITM3 alters CH25h enzymatic output, we analyzed the antiviral activity of conditioned supernatants from cells expressing IFITM3, CH25h, CH25h-M, or both proteins. To this end, we first performed a dose-response curve to soluble 25HC in HEK293T cells and assessed the effects on both cell toxicity and susceptibility to infection (Fig. A3A through C). Under our experimental conditions, we determined that cell toxicity was apparent only at concentrations of 25HC above 6.25 μM in this cell type (Figures A3A and B), while the susceptibility to infection of target cells incubated with different concentrations of soluble 25HC was inversely proportional to the concentration of 25HC and reached a plateau already at 0.195 μM (Fig. A3C).

**Fig 4:**
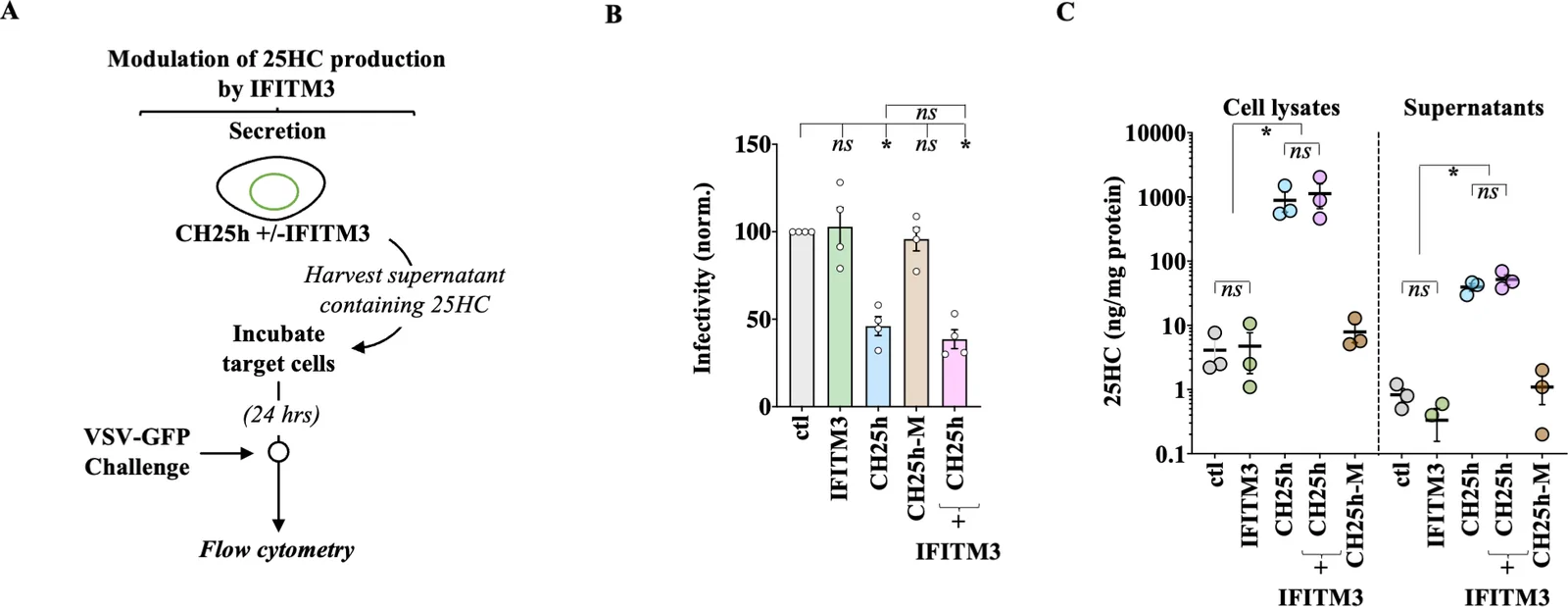
IFITM3 does not modify the extent of 25-hydroxycholesterol production and release from CH25h-producing cells. (**A**) Experimental scheme used here. (**B**) The supernatant of HEK293T cells expressing the indicated proteins was collected 24 h after transfection and used undiluted on untransfected cells for an additional 24 h. Cells were then challenged with VSV-GFP prior to flow cytometry analysis 18 h afterward. The graph presents AVG and SEM of four independent experiments. * and ns, *P* < 0.05 or nonsignificant, respectively, following ordinary one-way ANOVA and Tukey’s multiple comparisons tests between the indicated conditions. (**C**) HEK293T cells were transiently transfected with DNAs coding for the indicated DNAs, and 48 h later, both cells and supernatants were harvested and processed for 25HC quantification (23). The graph presents AVG and SEM of three independent experiments. * and ns, *P* < 0.05 or nonsignificant, respectively, following ordinary one-way ANOVA and Tukey’s multiple comparisons tests between the indicated conditions.

Next, to evaluate whether IFITM3 affected the levels of 25HC produced or secreted from cells expressing CH25h, the supernatant of cells was harvested 24 h after transfection, filtered to remove cellular debris, and directly used (with no dilution) to incubate nontransfected HEK293T cells. After an additional 24 h, the cells were challenged with VSV-GFP and then analyzed by fluorescence-activated cell sorting (FACS) to examine their susceptibility to infection. In this assay, therefore, 25HC secretion is measured indirectly through its effects on virion infectivity (Fig. 4A). Under these conditions, supernatants from CH25h-expressing cells—but not from CH25h-M or IFITM3 alone—conferred strong resistance to VSV when applied to naïve cells, indicating the presence of biologically active secreted 25HC (Fig. 4B). Importantly, supernatants from cells co-expressing IFITM3 and CH25h displayed antiviral activity indistinguishable from that displayed by CH25h alone, demonstrating that IFITM3 does not influence 25HC production by CH25h, or its secretion.

Finally, we measured the levels of 25HC in cells and supernatants of cells expressing CH25h alone or in combination with IFITM3 by mass spectroscopy as in reference 23 (Fig. 4C). 25HC could be readily detected in the supernatant and cell lysates from cells expressing active CH25h but not in the supernatant and lysates obtained from cells expressing the catalytically inactive mutant CH25h-M (average concentrations of 39.5 +/− 2.9 and 886 +/− 176.7 ng of 25HC per mg of total protein, for supernatants and cell lysates of WT CH25h, versus 1.1 +/− 0.3 and 7.9 +/− 1.4 ng of 25HC per mg of total protein, for supernatants and cell lysates of mutant CH25h, which represent the lower limit of detection of the assay). In both cases, the expression of IFITM3 did not alter the levels of 25HC measured under these conditions.

### The CH25h-25HC axis redistributes IFITM3 from endolysosomal compartments to the plasma membrane

**Fig 5:**
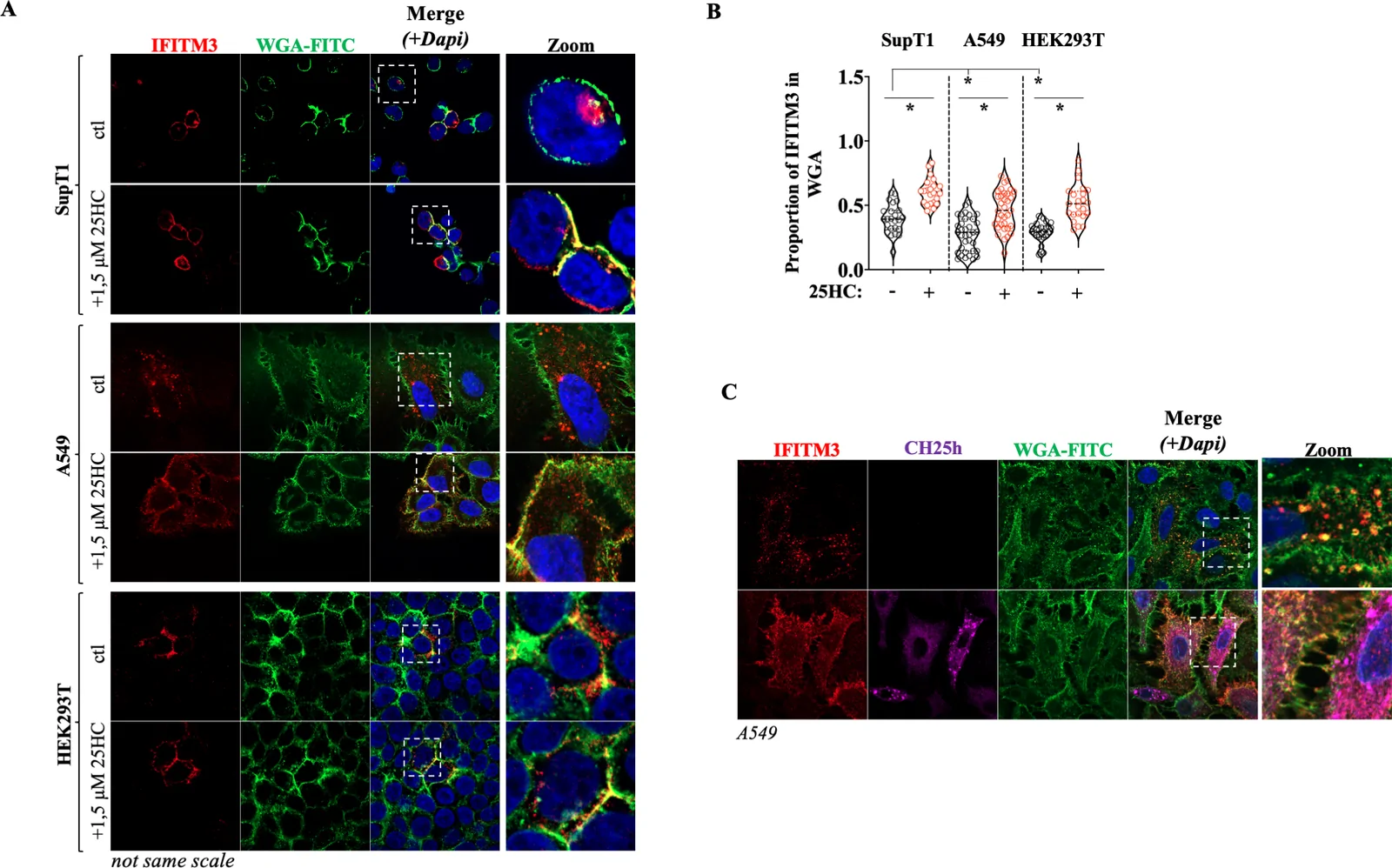
The CH25h-25HC axis promotes a redistribution of IFITM3 to the plasma membrane in several cell types. (**A**) Expression of IFITM3 was induced upon incubation with doxycycline in cell pools of SupT1, A549, and HEK293T obtained following retroviral-mediated gene transduction. Cells were further incubated or not with 25HC (1.5 μM) for 24 h prior to a 20-min labeling step at 37°C with FITC-WGA and confocal microscopy analysis. (**B**) The graph presents the quantification of the percentage of the IFITM3 signal in the FITC-WGA one (Mander’s coefficient) obtained in 4 to 5 independent experiments and in 22 to 35 individual cells per condition. *, *P* < 0.05, following one-way ANOVA and Tukey’s multiple comparison tests between the indicated conditions. (**C**) A549 cells stably expressing doxycycline-inducible forms of IFITM3 alone or together with CH25h were induced with doxycycline (3 μg/mL) for 24 h. Cells were then labeled with FITC-WGA as above, prior to confocal microscopy analysis.

We next asked whether CH25h and 25HC could instead influence IFITM3 behavior, and to address this issue, we examined the intracellular distribution of IFITM3 in cells exposed to high concentrations of 25HC (1.5 μM). Under these conditions, we observed a shift of IFITM3 from its canonical endosomal/vesicular pattern to the plasma membrane. Co-staining of cells with FITC-wheat germ agglutinin conjugates (WGA-FITC) confirmed a significant increase in plasma membrane-associated IFITM3 across multiple cell types (HEK293T, A549, and SupT1, Fig. 5A for representative pictures and Fig. 5B for quantitative analyses).

To reinforce this observation, we examined A549 cells stably co-expressing both IFITM3 and CH25h. As with exogenous 25HC treatment, CH25h expression induced a robust redistribution of IFITM3 toward the cell surface (Fig. 5C). Overall, these findings indicate that the 25HC-CH25h axis regulates the intracellular trafficking of IFITM3, independently from viral infection.

### 25HC reduces internalization of IFITM3 into early endosomes

**Fig 6:**
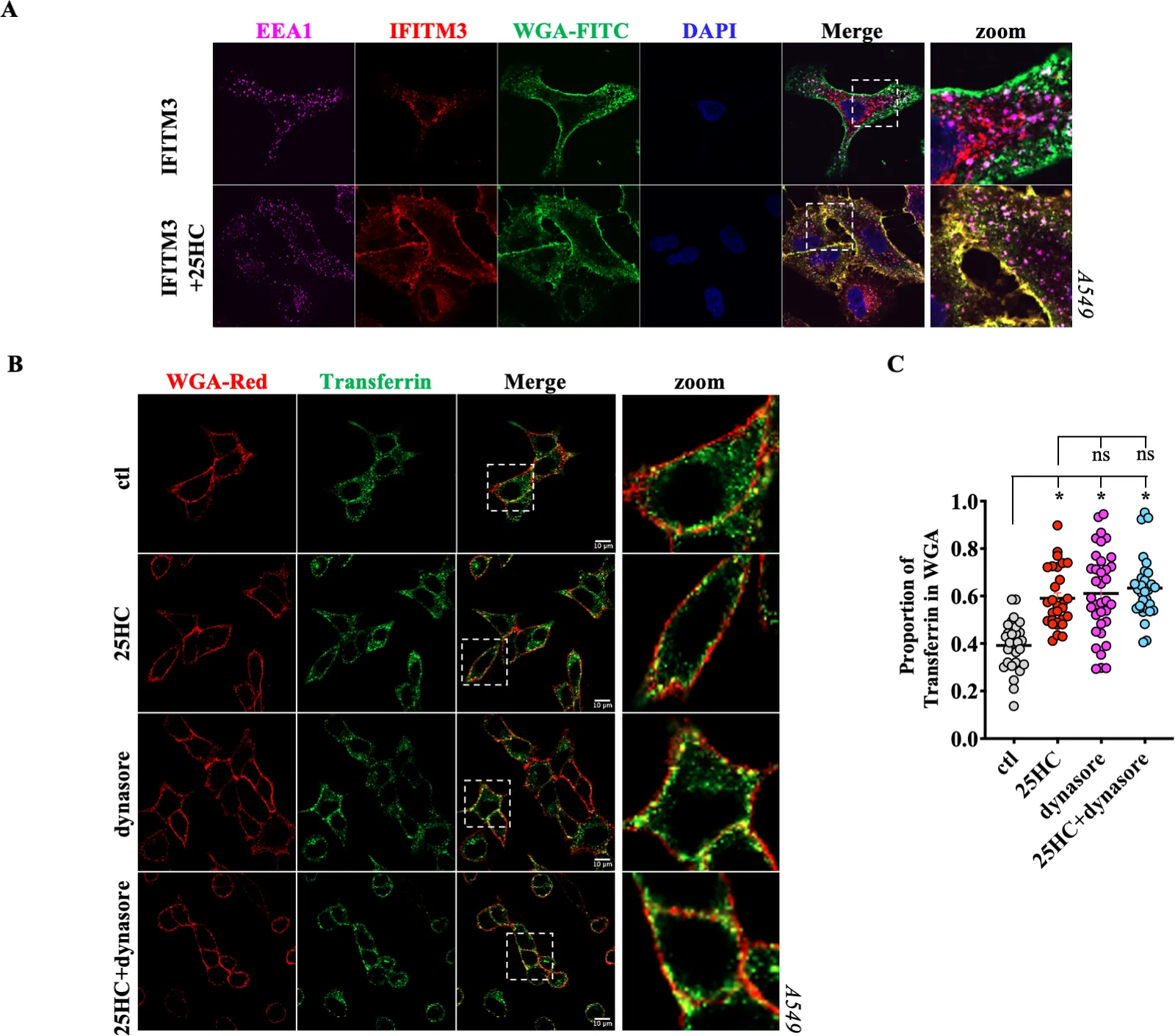
25HC leads to a decreased level of early endosomal recycling of IFITM3 from the plasma membrane. (**A**) Dox.-inducible IFITM3-expressing A549 cells were incubated for 24 h with dox. and/or 25HC prior to confocal microscopy analysis. (**B and C**) A549 cells were stimulated or not with 25HC as before. Prior to cell washing and cell processing for confocal microscopy analysis, when indicated, dynasore was added for 30 min at 37°C, while fluorescent Transferrin and WGA were added for the final 10 min at 37°C. Panel B presents representative results, while the graph in panel C presents the quantification of the percentage of the Transferrin signal in the WGA one in individual cells (Mander’s coefficient, in 24 to 36 individual cells per conditions; horizontal lines represent averages). * and ns, *P* < 0.05 or nonsignificant, respectively, following one-way ANOVA and Tukey’s multiple comparison tests between the indicated conditions.

Upon expression, IFITM3 is thought to reach the plasma membrane, where it is then endocytosed into early and then late endosomal vesicles thanks to specific sequences present in the long N-terminus of IFITM3 (24, 25). We, therefore, hypothesized that this step could be compromised by the 25HC-CH25h axis. To determine whether this was the case, we determined the amount of IFITM3 in early endosomes following confocal microscopy analysis in A549 cells exposed or not to 25HC. While when expressed alone, IFITM3 exhibited a consistent localization with the early endosomal marker EE1A, this colocalization was essentially lost in the presence of 25HC (Fig. 6A), therefore indicating that 25HC induces an early defect in the recycling of IFITM3 from the plasma membrane. To determine whether 25HC imposed a more general defect in endosomal trafficking, we examined the behavior of Transferrin, the ligand of the cellular Transferrin receptor (TfR), in the presence or absence of 25HC and dynasore, a well-known inhibitor of dynamin-dependent endocytosis. Prior to fixation and confocal microscopy analyses, cells were incubated for 10 min at 37°C with fluorescent Transferrin and WGA, and when indicated, dynasore was added 20 min prior to the addition of Transferrin/WGA. The proportion of plasma membrane Transferrin was then quantified upon colocalization with WGA by determining Mander’s coefficient. Under these conditions, we observed that the proportion of plasma membrane-associated Transferrin increased in a similar fashion when cells had been incubated with either 25HC or dynasore (Fig. 6B for representative results and Fig. 6C for quantification in 24 to 36 cells per condition). However, no differences were measured when cells were treated with both 25HC and dynasore. Overall, these results indicate that 25HC induces a general impairment in endocytosis that does not affect only IFITM3 but also the recycling of cellular receptors, such as the TfR.

Lack of additive effects of CH25h and IFITM3 cannot be explained by a CH25h-driven decrease in the antiviral activity of IFITM3 due to plasma membrane relocalization.

**Fig 7:**
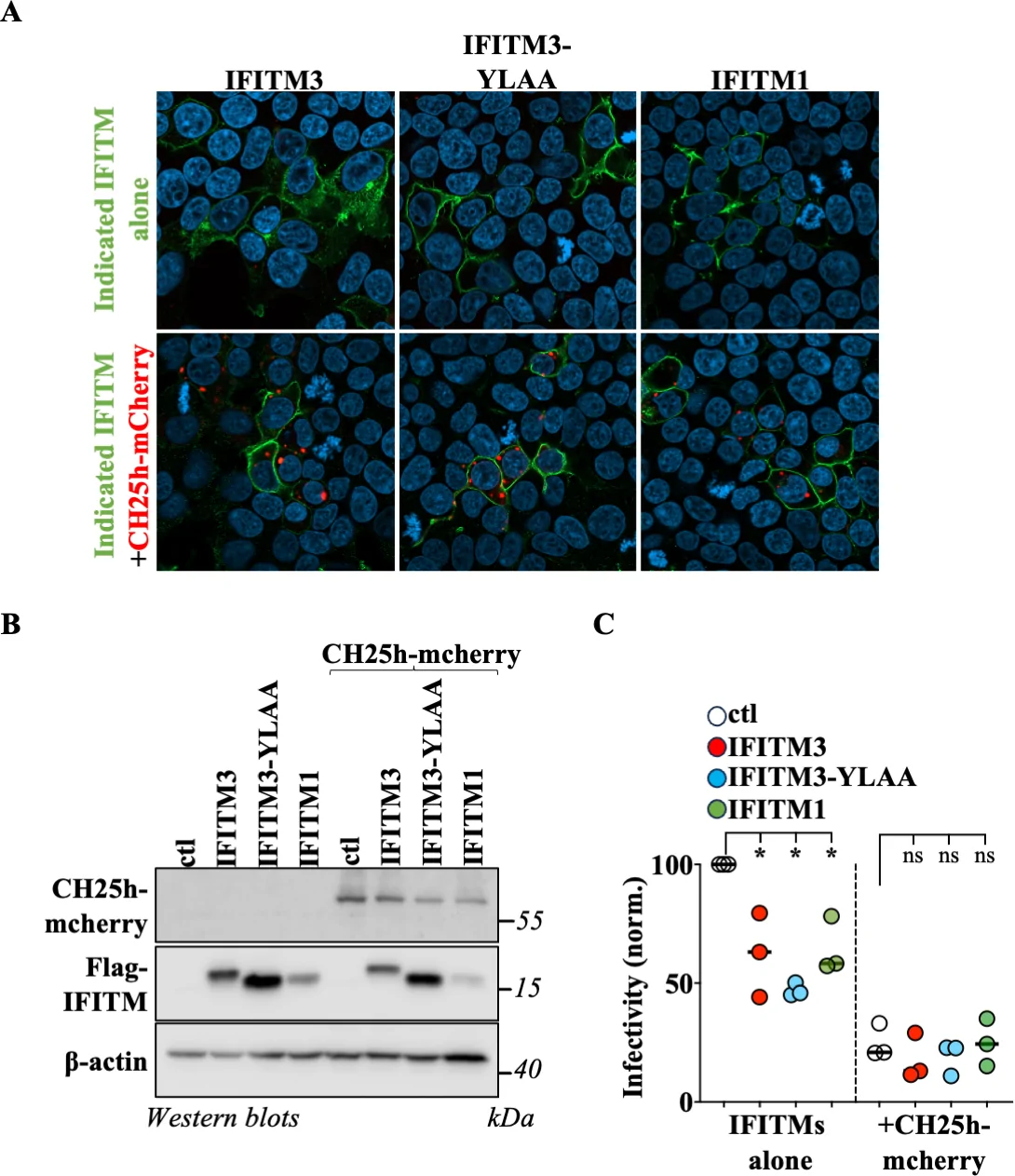
Plasma membrane IFITM proteins exhibit similar redundancy against VSV as IFITM3. A549 cells expressing wild-type IFITM3 in the presence or absence of CH25h were challenged with VSV and compared to cells expressing either wild-type IFITM1 or IFITM3-YLAA, a mutant in which the recruitment site for AP-2 has been mutated. (**A**) Confocal microscopy analysis of the indicated proteins. (**B**) WB analysis. (**C**) Susceptibility of the different cells to VSV infection as determined by flow cytometry. The panels present typical results obtained, while the graph presents the results of three independent experiments. ns and *, nonsignificant and *P* < 0.05, following one-way ANOVA and Tukey’s multiple comparison tests between the indicated conditions.

The antiviral behavior of IFITM3 is tributary of several parameters, among which is intracellular localization. To determine whether the lack of additive effects between IFITM3 and CH25h could be due to a decrease in the antiviral effects of plasma membrane-associated IFITM3, we compared IFITM3 to IFITM1, which is naturally distributed at the plasma membrane, and to an IFITM3-YLAA mutant (amino acids 20 to 23 bearing alanine substitutions in residues 22 and 23). This mutation prevents the recruitment of the AP-2 complex and results in an IFITM3 protein that remains at the plasma membrane (24, 26). Upon expression of these proteins alone or in combination with CH25h, cells were analyzed by confocal microscopy, WB, or challenged with VSV-GFP prior to flow cytometry analysis. As expected, IFITM1 and IFITM3-YLAA exhibited a strong localization at the plasma membrane that was not affected by co-expression with CH25h, and all proteins were clearly detectable upon WB analysis (Fig. 7A and B, respectively). When cells were challenged with VSV-GFP, IFITM3, IFITM1, and IFITM3-YLAA expression reduced viral infectivity to a similar extent, indicating that viral inhibition could be observed independently of the localization of the particular IFITM protein examined, at least under the experimental conditions used here (Fig. 7C). When these proteins were co-expressed with CH25h, no statistically significant differences in viral inhibition were observed when compared to CH25h alone, indicating that the lack of additive effects of CH25h and IFITM proteins on viral infectivity is not due to a loss of antiviral functions of IFITM proteins that relocalize at the plasma membrane.

### The CH25H-25HC axis enhances secretion of IFITM3 in exosome-like vesicles

**Fig 8:**
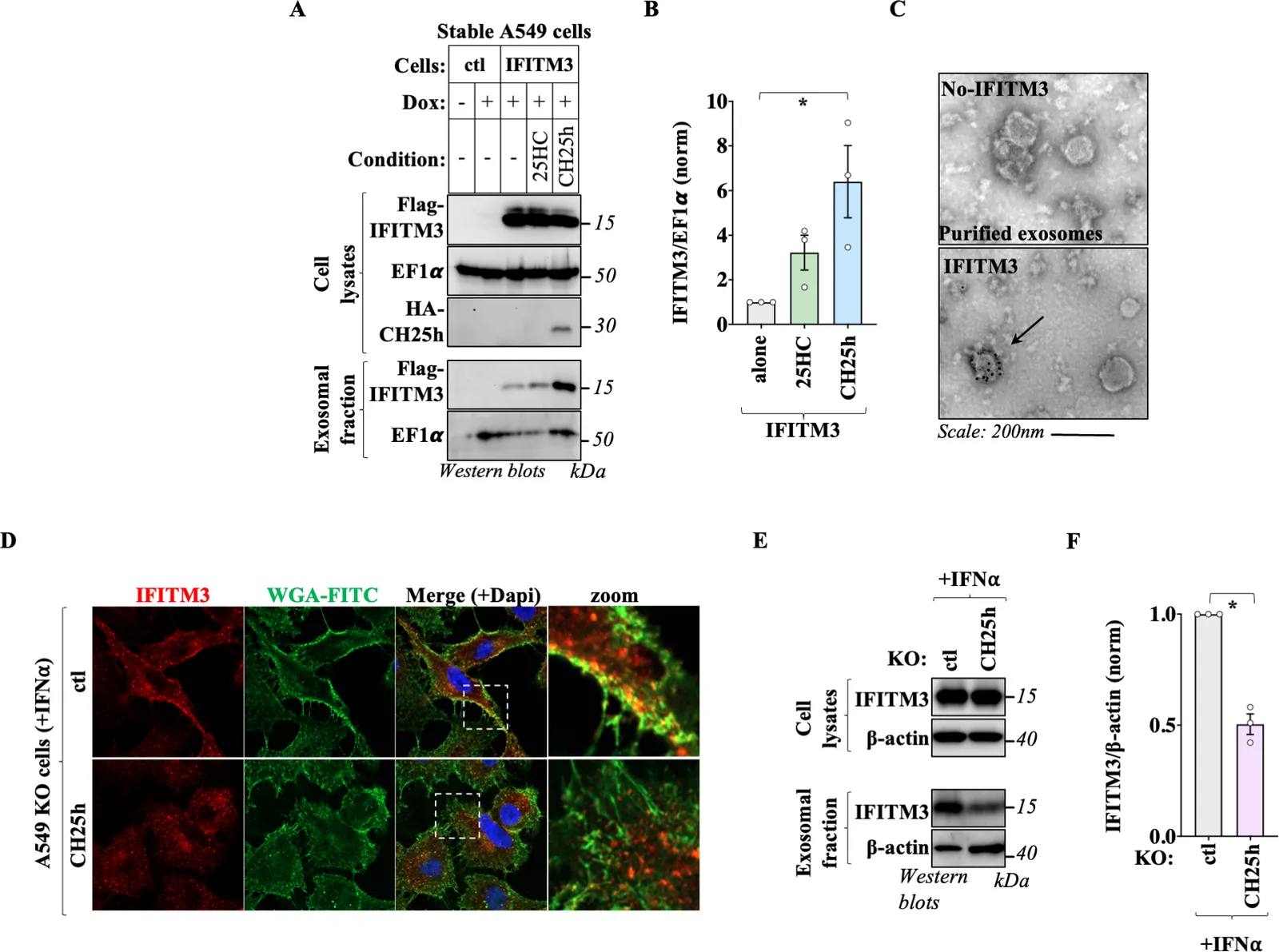
The CH25h-25HC axis leads to an increased accumulation of IFITM3 in exosomal vesicles. Single IFITM3 or double IFITM3/CH25h stable A549 cells were induced for 24 h with dox. prior to harvesting of both cell lysates and supernatants. When indicated, IFITM3 stable cells were either left untreated or co-stimulated with 25HC. After removal of cellular debris by syringe-filtering, exosomal vesicles were purified by ultracentrifugation through a 25% sucrose cushion, prior to WB analysis. The levels of IFITM3 present in the exosomal fraction were determined after densitometric quantification of the protein signals after WB analysis and following normalization for exosomal-associated levels EF1α or β-actin, as indicated. (**A**) Typical results obtained by WB. (**B**) AVG, SEM, and individual values of the levels of IFITM3 present in the exosomal fraction, following normalization for EF1α as quantified in three independent experiments. (**C**) Supernatants produced from control or IFITM3-expressing cells were purified as described before and were then examined by electron microscopy and immuno-gold labeling using an anti-Flag antibody. The panels present representative images obtained of over 400 vesicles scored, and the arrows point to typical IFITM3-positive exosomes. (**D–F**) Stable pools of A549 cell knockout for CH25h or control were stimulated for 48 h with IFNα (1,000 U/mL), prior to confocal microscopy (**D**), exosomal fraction purification, or WB analysis (**E**, representative result); a cumulative graph presenting the quantification of exosomal-IFITM3, following normalization for β-actin (**F**). *, *P* < 0.05, following an ordinary one-way ANOVA and Tukey’s multiple comparisons test between the indicated conditions (B) or a two-tailed Student’s *t* test (E).

IFITM3 has been previously detected in exosomal vesicles, in the absence of viral infection (27–29). We, therefore, tested whether its increased surface localization in the presence of 25HC or CH25h affects its release in extracellular vesicles. To this end, we purified exosomal vesicles from the supernatant of A549 cells expressing IFITM3 alone or in combination with CH25h or from cells expressing IFITM3 and incubated with 25HC. The supernatant was filtered and purified by ultracentrifugation through a 25% sucrose cushion, and pellets were then resuspended and analyzed by WB and densitometric quantification. The amount of IFITM3 present in the exosomal fraction was normalized for the amount of exosomal-associated EF1α or β-actin. Under these conditions, the levels of vesicle-associated IFITM3 increased when cells were treated with 25HC, and more strongly, upon coexpression of CH25h (3.2- versus 6.4-fold [Fig. 8A and B]). These findings indicate that CH25h promotes the extracellular release of IFITM3, likely as a downstream consequence of its altered trafficking. To demonstrate that IFITM3 is normally secreted upon association with exosomal vesicles, the supernatant of cells overexpressing IFITM3 was purified by ultracentrifugation through sucrose and then analyzed by immune-gold electron microscopy (Fig. 8C). Under these conditions, a readily detectable association of IFITM3 was observed with exosomal vesicles, in agreement with previous data in the literature indicating that, in the absence of viral infection, IFITM3 is a *bona fide* exosomal protein. Finally, to determine whether this could be observed under endogenous protein expression levels, we used retroviral-mediated gene transduction of CRISPR/Cas9 to generate pools of A549 cells knocked out for *CH25h* or control, and we examined the distribution of endogenous IFITM3 by confocal microscopy, as well as quantified the levels of endogenous IFITM3 in exosomal fractions following IFNα stimulation to induce endogenous IFITM3 and CH25h expression. In line with data obtained in overexpression experiments, the removal of CH25h in the context of IFN responses led to a decrease in the amount of plasma membrane-associated IFITM3 (Fig. 8D) and also led to a decrease in the IFITM3 levels associated with the exosomal fraction, in the absence of notable changes in its intracellular levels of expression (Fig. 8E for a representative result and Fig. 8F for quantification of three independent experiments). Overall, these results highlight that CH25h contributes to regulate the dynamics of IFITM3 in the cell and the levels of IFITM3 secreted outside the cell as exosomal fraction.

## DISCUSSION

In this study, we examined how two interferon-stimulated membrane-regulating antiviral factors, namely, IFITM3 and CH25h, interact functionally during infection with different enveloped viruses. Our analyses reveal that although both restrict viral entry with comparable qualitative outcomes, their antiviral activities are largely redundant during HIV-1 and VSV infection. Apparent synergistic effects were observed following spreading infection of HIV-1 in IFITM3-expressing SupT1 cells exposed to 25HC, but in light of the relatively long time span of the assay, we believe these effects are more likely due to an indirect effect of 25HC on the cell physiology rather than antiviral inhibition *per se*. A different situation was instead observed for orthoflaviviruses that, in our hands, exhibited little to no susceptibility to CH25h expression but remained highly sensitive to IFITM3. Although we have not investigated further the reasons for this behavior, we believe that the distinct membrane composition of orthoflaviviruses compared to *Rhabdoviridae* or *Retroviridae* may make them more resistant to cholesterol changes as driven by CH25h. In this respect, the maintenance of redundant mechanisms of viral inhibition targeting the same step provides a clear advantage for the cell against infection with a large spectrum of viruses.

A limitation of our study is that the functional connection between CH25h and IFITM3 has not been directly confirmed through viral challenges of double IFITM3/CH25h KO primary cells with an engaged interferon program. This is due to two reasons: first, the technical challenge of obtaining high levels of concurrent KO of two independent genes in primary cells; second, the complexity of the IFN program, which induces hundreds of genes that modulate viral infectivity. This context would have made it difficult to isolate and interpret the specific contributions of CH25h and IFITM3, which was instead possible thanks to the use of established cell lines that provide a more controlled setting.

Consistent with previous reports, both IFITM3 and CH25h potently inhibit HIV-1 and VSV infection by limiting the entry of virion particles into the cell, which represents the step of the viral life cycle most studied in the literature (4–8, 16, 18, 19, 30). To add to these reports, our results reveal that CH25h mediates a previously undescribed negative effect during the late phases of the HIV-1 life cycle. Although we have not characterized it in further detail here, this finding posits CH25h as the second antiviral factor capable of inhibiting the viral life cycle of HIV at both early and late phases. It is of interest that IFITM3 acts similarly at both early and late phases of the life cycle of different viruses and that both CH25h and IFITM3 act by altering the behavior of membranes.

In the case of CH25h and 25HC, the defect at the late phases of the viral life cycle may be due to an interference with cholesterol-rich virion assembly platforms essential for efficient budding and envelope incorporation (31) or else may be due to an indirect effect on envelope processing, as reported for the Lassa virus glycoprotein (32). Interestingly, a connection between lipid metabolism and IFITM3 has been suggested a few years ago, following the report of an interaction between IFITM3 and the vesicle-associated membrane protein (VAMP)-associated protein A (VAPA) (14). VAPA is an important ER adapter required for the transport of several classes of lipids from the ER to the Golgi (33). The IFITM3-VAPA interaction was reported to interfere with such transport, resulting in a specific increase in the intracellular levels of cholesterol, which could have explained the membrane-rigidifying behavior of IFITM3 (14). Despite the fact that this finding remains controversial (34, 35), the connection between CH25h and IFITM3 reopens the interesting possibility that a connection exists between the antiviral effects of IFITM3 and lipid metabolism.

A major and unexpected finding of our study is that the CH25h/25HC axis induces a robust redistribution of IFITM3 from endosomal compartments to the plasma membrane through a general modification of dynamin-dependent endosomal recycling. The relocalization of IFITM3 was observed with both exogenous 25HC as well as upon CH25h expression and occurred across multiple cell types. These changes do not affect the overall protection conferred by IFITM3 against viral infection because, under the experimental conditions used here, IFITM3 was as antiviral as IFITM proteins that were already concentrated at the plasma membrane. However, they lead to a strong increase in the amount of IFITM3 associated with secreted exosomal vesicles. This increase cannot be explained by differences in the expression levels of IFITM3, which remain steady, and can be more easily explained as a direct consequence of an accrued residence of IFITM3 at the plasma membrane.

While during viral infection we and others have demonstrated that IFITM3 can be readily incorporated into particles of a broad spectrum of viruses (9–11, 30), a few reports have indicated that IFITM3 can also be incorporated in exosomal vesicles in the absence of infection (27, 28). Our findings not only support these previous results but also extend them by indicating that the CH25h-25HC axis can significantly contribute to drive an increased presence of IFITM3 in this extracellular pool of protein. Even if secreted IFITM3 is unlikely to directly contribute to virus-to-cell membrane fusion in either target or virus-producing cells, we believe there are numerous ways in which this can contribute to antiviral responses at an intercellular scale. Exosomes are important mediators of cell-to-cell communication, and their content is important for the outcome of these communications in terms of cell activation status or cell polarization. A functional contribution of exosomal-associated IFITM3 has been reported in the transfer of an antiviral phenotype from one cell to the other (27, 28). Similarly, a recent study highlighted an important contribution of IFITM3 in the senescence-associated secretory phenotype (SASP) (27), a salient feature with which senescent cells influence their surrounding micro-environment. Given that senescence is associated with an inflammatory transcriptional signature and that CH25h is an integral part of this response, these results suggest the possibility that the CH25h-25HC axis drives, at least in part, the increased accumulation of IFITM3 in exosomal vesicles, thus contributing to the overall phenotypes associated with SASP and to the particular protein composition of SASP vesicles.

Altogether, our findings highlight the membrane as an important point of convergence for viral inhibition by two broad antiviral factors, IFITM3 and CH25h. These factors do not exert additive effects on viral inhibition and appear, thus, largely redundant in the case of VSV and HIV-1 infection because they are susceptible to both CH25h and/or IFITM3. However, in the case of the orthoflaviviruses tested here, IFITM3 compensates for an apparent lack of susceptibility to CH25h. Thus, this result illustrates why it is advantageous for the cell to maintain two effectors that target the same step in the life cycle of several classes of viruses: to provide coverage against viruses that have evolved resistance to one of them. Beyond this functional complementarity, our results also indicate that IFITM3 and CH25h are not completely independent from each other. Indeed, the CH25h/25HC axis modifies the localization of IFITM3, driving higher levels of IFITM3 at the plasma membrane and in exosomal fractions. Thus, although the combined action of CH25h and IFITM3 plays no additive effects at the cell-intrinsic level, it can contribute to antiviral immune responses by influencing, in a paracrine manner, how cells communicate with each other.

## MATERIALS AND METHODS

### Cell culture, DNA constructs, antibodies, densitometry, and compounds

Human embryonic kidney and human lung epithelial cells (HEK293T and A549, ATCC catalog numbers: CRL-3216 and CCL-185, respectively) were cultured in complete Dulbecco’s modified Eagle medium (DMEM) with 10% fetal calf serum (FCS) (Sigma catalog number: F7524), while the T cell line SupT1 (ATCC catalog number: CRL-1942) was cultured in complete RPMI-1640 media also supplemented with 10% FCS.

Flag-tagged IFITM3 expressed either from a CMV promoter or in a doxycycline-inducible form in the context of the murine leukemia virus (MLV)-derived retroviral vector have been previously described (11). Flag-tagged IFITM1 has been also previously described (11). The YLAA mutation was introduced in the coding sequence of IFITM3 (amino acids 20–24) by standard molecular biology techniques. A codon-optimized version of the CH25h was fused at the C terminus to mcherry in the context of an HIV-1-based lentiviral vector (11). A catalytically inactive mutant containing the four histidine residues at positions 242, 243, 246, and 247 of the catalytic site of CH25h changed to glutamic acid was similarly constructed by gene synthesis (Genewiz). A non-mCherry HA-tagged N-terminal version was generated by standard molecular biology in the pRetroX-Tight-Puro vector. pcDNA-D1ER coding an ER-targeted enhanced CFP was a gift from Amy Palmer & Roger Tsien (catalog number: 36325; Addgene [36]).

HIV-1 proviral clone NL4-3 was obtained through the AIDS reagents and repository of the NIH, USA.

Single-round infection-competent HIV-1-based lentiviral vectors were produced upon co-transfection of four different plasmids: HIV-1 Rev, NL4-3 Env, Gag-Pro-Pol, and mini-viral genome coding for a GFP reporter (described in reference 10, see below for the procedure).

Pools of cells expressing in a stable manner IFITM3 and/or CH25h were obtained after transduction with an MLV retroviral-based gene transduction system pRetroX-Tight system (Clontech), using the following plasmids: Gag-Pro-Pol of MLV (TG5349); the VSV-G envelope; MLV-based genomes coding IFITM3, CH25h, or the transcriptional transactivator rtTA (as described in reference 11, see below for the procedure).

The following primary antibodies were used for WB or confocal microscopy, as indicated: mouse monoclonal antibodies: anti-α-tubulin, anti-HA, anti-β-actin (catalog numbers: T5168, H3663, and A1978; Sigma-Aldrich, respectively), anti-EF1α (catalog number: CBP-KK1; Millipore), and anti-EEA1 (catalog number: MA5-14794; Life Technologies); rabbit polyclonal antibodies: anti-IFITM3 (catalog number: 11714-1-AP; Proteintech), anti-FLAG (catalog number: Ab52649; Abcam), and anti-mcherry (catalog number: Ab52649; Abcam).

The following secondary antibodies were used for WB: anti-mouse and anti-rabbit IgG-peroxidase-conjugated (Sigma, St. Louis, MO, USA, catalog numbers: A9044 and AP188P). The following were used for confocal microscopy: donkey anti-rabbit IgG conjugated with Alexa Fluor 647 or 555, donkey anti-mouse IgG-Alexa Fluor 488 conjugate, and donkey anti-Goat IgG-Alexa Fluor 594 (Life Technologies, Carlsbad, USA, catalog numbers: A-21207, or A-32795 or A-32974, A-21202, and A-11058).

Wheat germ agglutinin conjugated to FITC (FITC-WGA) was purchased from Santa Cruz Biotechnology (catalog number: sc-18822); 25HC was purchased from SIGMA (catalog number: H1015). Cell viability in different concentrations of 25HC was evaluated by cell counting upon trypan blue exclusion at different days post-treatment.

Following WB, the amount of exosomal-associated IFITM3 was quantified by densitometry analysis using the Chemidoc Imaging System (Bio-Rad), following normalization for the levels of EF1α or β-actin.

### Production of viruses and infections

Single-round infection-competent HIV-1 used to study the early phases of HIV-1 infection was produced by calcium phosphate DNA transfection of HEK293T cells (Gag-Pro-Pol packaging construct, mini-viral genome, Rev, and the NL4-3 HIV-1 envelope: 8:8:0.5:2 μg each per 10-cm dish). The supernatants were collected 48 h afterward and syringe-filtered to remove cellular debris. Virions were purified through a 25% (wt/vol) sucrose cushion for 1 h 30 min at 28,000 rpm. The number of infectious viral particles was determined by infecting HeLa cells with virus dilutions and by quantifying the number of GFP-positive cells obtained 2 to 3 days after by flow cytometry. The infectious titer equivalent of non-GFP coding viruses was determined against standards of known infectivity by exo-RT (11). A similar setup was used for the production of MLV-based vectors (37).

The early phases of HIV-1 infection were analyzed by challenging target cells expressing IFITM3 +/− CH25h at multiplicities of infection (MOIs) between 0.5 and 1 and by quantifying the number of GFP-positive cells 2 to 3 days post-infection by flow cytometry.

The late phases of the HIV-1 life cycle were analyzed as follows. Virion particles were produced in HEK293T cells co-transfected with plasmids coding the HIV-1 proviral DNA (NL4-3) along with CH25h-mCherry alone or in combination with Flag-IFITM3. After 48 h, the virus was harvested and used to challenge TZM-BL reporter cells. The amount of infectious virion particles was quantified after luciferase analysis on cell lysates using the BrightGlow Lysis Reagent (Promega E2620) and the Tecan Spark Luminometer. Infectious virus yields under various conditions were consistently expressed as fold-change compared with paired viral infection conditions in the absence of IFITM3 or CH25h.

Spreading infections with replication-competent NL4-3 viruses in SupT1 cells were also assessed by intracellular p24 assays and flow cytometry. Cells were collected at the indicated time points, fixed, and permeabilized using the FIX & PERM Cell Permeabilization Kit (catalog number: GAS003, Life Technologies) and stained for intracellular p24 using an RD1-conjugated anti-HIV-1 core antigen antibody (clone KC57; catalog number: 6604667, Beckman Coulter) prior to FACS analysis.

Replication-competent GFP-coding VSV-Indiana serotype Rhabdovirus (VSV-GFP) bearing an additional transcription cassette between M and G was used to assess the role of the indicated proteins and conditions on VSV spreading infections. In this case, infectious units were calculated based on infections carried out with limiting dilutions of the virus stock followed by flow cytometry analysis 18 h post-infection.

Infections were carried out with MOIs between 0.1 and 1. The percentage of infected cells was determined at 18 h post-infection by flow cytometry on a FACS Canto II (Becton Dickinson, USA).

The following orthoflaviviruses were used: dengue virus (DENV-4, Indonesia) and Zika (Asian lineage, accession number: KU955592) at an MOI of 0.5 and West Nile virus (WNV, lineage 2, Austria, accession number: KF179640) at an MOI of 0.1. The extent of infection was determined 24 h later following fixation of trypsinized cells for 15 min with medium A (FIX & PERM(R) kit, Invitrogen, GAS002) and staining with both primary and secondary antibodies in medium B on ice for 30 min (anti pan-envelope (D1-4G2-4-15 (4G2), Bio-Techne, 0.5 µg/mL) and secondary donkey anti-rabbit Alexa Fluor 488 antibody, 2 µg/mL). After washing, cells were analyzed by flow cytometry.

### Generation of stable cell lines

Doxycycline-inducible stable cells overexpressing Flag-IFITM3 and/or HA-CH25h were generated using the pRetroX-Tight system (Clontech). Briefly, MLV retroviral vectors are produced by calcium phosphate DNA transfection of HEK293T cells with plasmids coding the MLV Gag-Pro-Pol, the VSVg envelope, and two pRetro-X based mini-viral genomes: the first coding the protein of interest (Flag-IFITM3 and/or HA-CH25h) under the control of the doxycycline-inducible promoter and the second coding the transcriptional transactivator rtTA (ratio 8:4:4:4 for a total of 20 μg per 10-cm dish). Virion particles in the cell supernatant were directly used for cell transduction followed by selection of cell pools thanks to the Puromycin (Sigma, catalog number: P8833) and G418 (Sigma, catalog number: G8168) resistance genes carried by the two constructs. Cell pools were maintained as such to avoid clone-dependent variations.

Genetic ablation of CH25h was obtained following three rounds of HIV-mediated gene transduction of A549 cells with Cas9 and CRISPR-bearing HIV-1-based lentiviral vectors (gift of Feng Zhang, obtained from Addgene catalog numbers: 52963 and 52962). Selection of pools of control-KO or CH25h-KO cells was obtained following blasticidin (Invivogen, catalog number: ant-bl-1) and puromycin (Sigma, catalog number: P8833) selection thanks to the resistance genes coded by the two vectors. Guide sequences were as follows: CH25h-1 TCGACTTTTCACAGGACTCA (chr10:89207336–89207358); CH25h-2 CTTCTCCATCACCACATAC (chr10:89207145–89207167); control-1 TCATGCTTGCTTGGGCAAAA; control-2 GACCTTCATTGAAGAAAAGC. Gene editing was controlled following PCR amplification of genomic DNA using primers AGGGATGGAAAGGCAGCTGAC and GAGCACAGGATATCCAGGAC, by cloning of the corresponding PCR fragment and by sequencing of ten individual clones per condition. To avoid cell derivation over time, stable cells were routinely generated.

### HIV-1 entry assay according to the entry/uncoating assay based on core-packaged RNA availability and translation system (EURT)

This assay is based on the direct translation of a luciferase-bearing HIV-1 genome mimic that can be directly translated in the cell, yielding a precise measure of virus entry into the cell cytoplasm (21). Single round of infection NL4-3 Envelope-bearing HIV-1 viruses incorporating this reporter (EU-repRNA) were produced by transient transfection of HEK293T cells, as described above. Infections were carried out for 18 h in the presence of the capsid destabilizing compound PF74 (10 μg/mL, Sigma, catalog number: SML0835-5), prior to cell lysis and luciferase activity quantification (Promega, catalog number: E2620, according to the manufacturers’ instructions).

### VSV entry (RT-qPCRs)

VSV entry was measured by quantifying the levels of viral RNA in cells 20 min after infection by RT-qPCR in the presence of 100 μg/mL of cycloheximide (CHX, Sigma, catalog number: 4859) to prevent the translation of viral proteins from positive-strand viral RNAs that ignite the full amplification of viral RNA. Prior to cell lysis, cells were washed and treated for 10 min at 37°C with trypsin to remove non-internalized virions. Total cellular RNA was extracted according to the manufacturer’s instructions (Macherey-Nagel NucleoSpin RNA catalog number: 740956), and reverse transcription was performed using random hexamers and oligo(dT) with the SuperScript III reverse transcriptase (Thermo Fisher catalog number: 18080), also following the manufacturers’ instructions. qPCRs were performed on a StepOne Plus real-time PCR system (Applied Biosystems) using the FastStart universal SYBR green master mix (Roche Diagnostics, catalog number: 4913914001). Primers amplified the GFP gene inserted into the viral genome: GAACGGCATCAAGGTGAACT and TGCTCAGGTAGTGGTTGTCG.

### Preparation/analysis of exosomal fractions

Stably transduced A549 cells were seeded at equal density and allowed to adhere overnight. The following day, the medium was replaced with or without fresh DMEM containing doxycycline (2 µg/mL) to induce transgene expression, with or without 25-HC at a final concentration of 1.5 µM. After 48 h of induction, culture supernatants were harvested, clarified by low-speed centrifugation, and layered onto 25% (wt/vol) sucrose cushions. Samples were ultracentrifuged at 28,000 rpm for 1.5 h at 4°C in an SW41Ti rotor. The resulting pellets containing an enriched exosomal fraction were resuspended directly in 2× SDS loading buffer for SDS-PAGE and further analyzed by SDS-PAGE gel followed by Western blotting. The amount of IFITM3 protein present in this fraction was determined following densitometric quantification of the corresponding bands (Fiji) and normalization for either EF1α or β-actin present in the same exosomal fraction.

### Immunogold labeling and electron microscopy

Formvar-/carbon-coated nickel grids were deposited on a drop of purified, unfixed exosomal preparations produced in the presence or absence of Flag-tagged IFITM3 for 5 min, followed by incubation with an anti-Flag antibody (F7425, Sigma, St. Louis, MO, USA), then by incubation with a 1:30 gold-conjugated (10 nm) goat-anti-Rabbit IgG (Aurion, Wageningen, Netherlands), and fixation in 1% glutaraldehyde. Negative staining was performed using 2% uranyl acetate (Agar Scientific, Stansted, UK), followed by transmission electron microscope analyses (JEOL 1011, Tokyo, Japan).

### Confocal microscopy

Cells were seeded on glass coverslips coated with poly-L-Lysine 0.01% (Sigma cat. P4832) after Lipofectamine 3000-mediated DNA transfection, according to the manufacturer’s instructions (catalog number: L3000008, Invitrogen). Twenty-four hours later, cells were fixed with PFA (4%), then permeabilized with 0.1% Triton X-100 in PBS, and then incubated with the indicated primary and secondary antibodies. Coverslips were stained with a DAPI-containing solution (ThermoFisher catalog number: 62248) and mounted using the anti-quenching solution Fluormount G (Southern Biotech). Fluorescent confocal images were acquired on a Zeiss LSM 980 AiryScan confocal microscope. Pictures were analyzed with the ImageJ software. For quantification purposes, cells were incubated with Transferrin-CF448 (Ozyme catalog number: BTM00081, final concentration of 25 μg/mL) and wheat germ agglutinin (WGA-red, Invitrogen catalog number: I6500-66, final concentration of 3 μg/mL) for 10 min at 37°C prior to cell washing and fixation. For each cell, membrane association was measured by determining the amounts of IFITM3, or Transferrin, signal associated with WGA (Mander’s coefficient, Fiji). When indicated, dynasore (Sigma catalog number: D7693) was added at a final concentration of 160 μg/mL for 30 min at 37°C total (i.e., 20 min prior to addition of Transferrin/WGA).

### 25HC measurement

HEK293T cells were transiently transfected by calcium phosphate in 10-cm dishes with plasmids encoding the indicated proteins for a total of 20 μg DNA per dish. At 48 h post-transfection, both cells and culture supernatants were collected for 25HC measurement. 25HC in cell homogenates and supernatants was extracted with a solvent mixture containing tert-butyl methyl ether/methanol 10/3 (vol/vol), spiked with epicoprostanol as the internal standard. Lipids were partitioned in the upper phase after the addition of saline and saponified by methanolic potassium hydroxide (0.5 N, 60°C, 15 min). The fatty acids released were methylated with BF3-methanol (12%, 60°C, 15 min), in order to not interfere with the chromatography of sterols. The 25HC was re-extracted in hexane and silylated, as described previously (23). The trimethylsilylether derivatives of 25HC were separated by gas chromatography (GC) (Hewlett–Packard 6890 series) in a medium-polarity capillary column RTX-65, (65% diphenyl/35% dimethyl polysiloxane, length 30 m, diameter 0.32 mm, and film thickness 0.25 µm) (Restesk, Evry, France). The mass spectrometer (Agilent 5975 inert XL, Agilent, Santa Clara, CA, USA) in series with the GC was set up for the detection of positive ions. Ions were produced in the electron impact mode at 70 eV. 25OHC was identified by the fragmentogram in the scanning mode and quantified by selective monitoring of the specific ions after normalization with the internal standard epicoprostanol and in calibration with weighed standards.

### Statistical analyses

Two-tailed Student’s *t* tests or one-way analysis of variance (ANOVA) tests were calculated using the Prism 8 software, as specified in the legend to figures.

### Software

The following software was used: for confocal microscopy, Fiji software (version 2.0.0), Zen (version 2.3, Zeiss), and Imaris 9.2.0 software (Oxford Instruments Group); for WB, Image Lab Touch Software (version 2.0.0.27, Chemidoc Imaging System from Bio-Rad); and for flow cytometry, FlowJo (version 10.10, BD).

Statistics and graphs used were as follows: GraphPad Prism 8 (8.4.3, GraphPad Software, LLC) and Excel (16.16.3, Microsoft).

## Data Availability

All relevant data are within the paper.

## SUPPLEMENTAL MATERIAL

Data Set S1 jvi.01378-26-s0001.xlsx Raw data.

Supplemental figures jvi.01378-26-s0002.docx Fig. A1 to A3.

ASM does not own the copyrights to Supplemental Material that may be linked to, or accessed through, an article. The authors have granted ASM a non-exclusive, world-wide license to publish the Supplemental Material files. Please contact the corresponding author directly for reuse.

## References

[1] Gómez-Herranz M, Taylor J, Sloan RD. 2023. IFITM proteins: understanding their diverse roles in viral infection, cancer, and immunity. J Biol Chem 299:102741. doi:10.1016/j.jbc.2022.102741 PMC9800550 36435199

[2] Marziali F, Cimarelli A. 2021. Membrane interference agaiy intrinsic antiviral factors: the case ofnst HIV-1 by intrinsic antiviral factors: the case of IFITMs. Cells 10:1171. doi:10.3390/cells10051171 PMC8151167 34065027

[3] Wang J, Luo Y, Katiyar H, Liang C, Liu Q. 2024. The antiviral activity of interferon-induced transmembrane proteins and virus evasion strategies. Viruses 16:734. doi:10.3390/v16050734 PMC11125860 38793616

[4] Brass AL, Huang I-C, Benita Y, John SP, Krishnan MN, Feeley EM, Ryan BJ, Weyer JL, van der Weyden L, Fikrig E, Adams DJ, Xavier RJ, Farzan M, Elledge SJ. 2009. The IFITM proteins mediate cellular resistance to influenza A H1N1 virus, West Nile virus, and dengue virus. Cell 139:1243–1254. doi:10.1016/j.cell.2009.12.017 PMC2824905 20064371

[5] Spence JS, He R, Hoffmann HH, Das T, Thinon E, Rice CM, Peng T, Chandran K, Hang HC. 2019. IFITM3 directly engages and shuttles incoming virus particles to lysosomes. Nat Chem Biol 15:259–268. doi:10.1038/s41589-018-0213-2 PMC6466627 30643282

[6] Guo X, Steinkühler J, Marin M, Li X, Lu W, Dimova R, Melikyan GB. 2021. Interferon-induced transmembrane protein 3 blocks fusion of diverse enveloped viruses by altering mechanical properties of cell membranes. ACS Nano 15:8155–8170. doi:10.1021/acsnano.0c10567 PMC8159881 33656312

[7] Suddala KC, Lee CC, Meraner P, Marin M, Markosyan RM, Desai TM, Cohen FS, Brass AL, Melikyan GB. 2019. Interferon-induced transmembrane protein 3 blocks fusion of sensitive but not resistant viruses by partitioning into virus-carrying endosomes. PLoS Pathog 15:e1007532. doi:10.1371/journal.ppat.1007532 PMC6347298 30640957

[8] Klein S, Golani G, Lolicato F, Lahr C, Beyer D, Herrmann A, Wachsmuth-Melm M, Reddmann N, Brecht R, Hosseinzadeh M, Kolovou A, Makroczyova J, Peterl S, Schorb M, Schwab Y, Brügger B, Nickel W, Schwarz US, Chlanda P. 2023. IFITM3 blocks influenza virus entry by sorting lipids and stabilizing hemifusion. Cell Host Microbe 31:616–633. doi:10.1016/j.chom.2023.03.005 37003257

[9] Compton AA, Bruel T, Porrot F, Mallet A, Sachse M, Euvrard M, Liang C, Casartelli N, Schwartz O. 2014. IFITM proteins incorporated into HIV-1 virions impair viral fusion and spread. Cell Host Microbe 16:736–747. doi:10.1016/j.chom.2014.11.001 PMC7104936 25464829

[10] Tartour K, Appourchaux R, Gaillard J, Nguyen XN, Durand S, Turpin J, Beaumont E, Roch E, Berger G, Mahieux R, Brand D, Roingeard P, Cimarelli A. 2014. IFITM proteins are incorporated onto HIV-1 virion particles and negatively imprint their infectivity. Retrovirology 11:103. doi:10.1186/s12977-014-0103-y PMC4251951 25422070

[11] Tartour K, Nguyen XN, Appourchaux R, Assil S, Barateau V, Bloyet LM, Burlaud Gaillard J, Confort MP, Escudero-Perez B, Gruffat H, et al.. 2017. Interference with the production of infectious viral particles and bimodal inhibition of replication are broadly conserved antiviral properties of IFITMs. PLoS Pathog 13:e1006610. doi:10.1371/journal.ppat.1006610 PMC5619827 28957419

[12] Lee J, Robinson ME, Ma N, Artadji D, Ahmed MA, Xiao G, Sadras T, Deb G, Winchester J, Cosgun KN, et al.. 2020. IFITM3 functions as a PIP3 scaffold to amplify PI3K signalling in B cells. Nature 588:491–497. doi:10.1038/s41586-020-2884-6 PMC8087162 33149299

[13] Rahman K, Coomer CA, Majdoul S, Ding SY, Padilla-Parra S, Compton AA. 2020. Homology-guided identification of a conserved motif linking the antiviral functions of IFITM3 to its oligomeric state. eLife 9:e58537. doi:10.7554/eLife.58537 PMC7665892 33112230

[14] Amini-Bavil-Olyaee S, Choi YJ, Lee JH, Shi M, Huang IC, Farzan M, Jung JU. 2013. The antiviral effector IFITM3 disrupts intracellular cholesterol homeostasis to block viral entry. Cell Host Microbe 13:452–464. doi:10.1016/j.chom.2013.03.006 PMC3646482 23601107

[15] Lund EG, Kerr TA, Sakai J, Li WP, Russell DW. 1998. cDNA cloning of mouse and human cholesterol 25-hydroxylases, polytopic membrane proteins that synthesize a potent oxysterol regulator of lipid metabolism. J Biol Chem 273:34316–34327. doi:10.1074/jbc.273.51.34316 9852097

[16] Liu SY, Aliyari R, Chikere K, Li G, Marsden MD, Smith JK, Pernet O, Guo H, Nusbaum R, Zack JA, Freiberg AN, Su L, Lee B, Cheng G. 2013. Interferon-inducible cholesterol-25-hydroxylase broadly inhibits viral entry by production of 25-hydroxycholesterol. Immunity 38:92–105. doi:10.1016/j.immuni.2012.11.005 PMC3698975 23273844

[17] Hannedouche S, Zhang J, Yi T, Shen W, Nguyen D, Pereira JP, Guerini D, Baumgarten BU, Roggo S, Wen B, et al.. 2011. Oxysterols direct immune cell migration via EBI2. Nature 475:524–527. doi:10.1038/nature10280 PMC4297623 21796212

[18] Li C, Deng Y-Q, Wang S, Ma F, Aliyari R, Huang X-Y, Zhang N-N, Watanabe M, Dong H-L, Liu P, et al.. 2017. 25-Hydroxycholesterol protects host against ZIKA virus infection and its associated microcephaly in a mouse model. Immunity 46:446–456. doi:10.1016/j.immuni.2017.02.012 PMC5957489 28314593

[19] Gomes B, Gonçalves S, Disalvo A, Hollmann A, Santos NC. 2018. Effect of 25-hydroxycholesterol in viral membrane fusion: insights on HIV inhibition. Biochim Biophys Acta Biomembr 1860:1171–1178. doi:10.1016/j.bbamem.2018.02.001 29408450

[20] Yuan Y, Wang Z, Tian B, Zhou M, Fu ZF, Zhao L. 2019. Cholesterol 25-hydroxylase suppresses rabies virus infection by inhibiting viral entry. Arch Virol 164:2963–2974. doi:10.1007/s00705-019-04415-6 31552533

[21] Da Silva Santos C, Tartour K, Cimarelli A. 2016. A novel entry/uncoating assay reveals the presence of at least two species of viral capsids during synchronized HIV-1 infection. PLoS Pathog 12:e1005897. doi:10.1371/journal.ppat.1005897 PMC5045187 27690375

[22] Ostertag D, Hoblitzell-Ostertag TM, Perrault J. 2007. Cell-type-specific growth restriction of vesicular stomatitis virus polr mutants is linked to defective viral polymerase function. J Virol 81:492–502. doi:10.1128/JVI.01217-06 PMC1797469 17065214

[23] Segala G, David M, de Medina P, Poirot MC, Serhan N, Vergez F, Mougel A, Saland E, Carayon K, Leignadier J, et al.. 2017. Dendrogenin A drives LXR to trigger lethal autophagy in cancers. Nat Commun 8:1903. doi:10.1038/s41467-017-01948-9 PMC5712521 29199269

[24] Jia R, Xu F, Qian J, Yao Y, Miao C, Zheng YM, Liu SL, Guo F, Geng Y, Qiao W, Liang C. 2014. Identification of an endocytic signal essential for the antiviral action of IFITM3. Cell Microbiol 16:1080–1093. doi:10.1111/cmi.12262 PMC4065222 24521078

[25] Chesarino NM, McMichael TM, Yount JS. 2015. E3 ubiquitin ligase NEDD4 promotes influenza virus infection by decreasing levels of the antiviral protein iFITM3. PLoS Pathog 11:e1005095. doi:10.1371/journal.ppat.1005095 PMC4532365 26263374

[26] Chesarino NM, McMichael TM, Hach JC, Yount JS. 2014. Phosphorylation of the antiviral protein interferon-inducible transmembrane protein 3 (IFITM3) dually regulates its endocytosis and ubiquitination. J Biol Chem 289:11986–11992. doi:10.1074/jbc.M114.557694 PMC4002105 24627473

[27] Borghesan M, Fafián-Labora J, Eleftheriadou O, Carpintero-Fernández P, Paez-Ribes M, Vizcay-Barrena G, Swisa A, Kolodkin-Gal D, Ximénez-Embún P, Lowe R, Martín-Martín B, Peinado H, Muñoz J, Fleck RA, Dor Y, Ben-Porath I, Vossenkamper A, Muñoz-Espin D, O’Loghlen A. 2019. Small extracellular vesicles are key regulators of non-cell autonomous intercellular communication in senescence via the interferon protein IFITM3. Cell Rep 27:3956–3971. doi:10.1016/j.celrep.2019.05.095 PMC6613042 31242426

[28] Zhu X, He Z, Yuan J, Wen W, Huang X, Hu Y, Lin C, Pan J, Li R, Deng H, Liao S, Zhou R, Wu J, Li J, Li M. 2015. IFITM3-containing exosome as a novel mediator for anti-viral response in dengue virus infection. Cell Microbiol 17:105–118. doi:10.1111/cmi.12339 PMC7162390 25131332

[29] Hrdinova T, Toman O, Dresler J, Klimentova J, Salovska B, Pajer P, Bartos O, Polivkova V, Linhartova J, Machova Polakova K, Kabickova H, Brodska B, Krijt M, Zivny J, Vyoral D, Petrak J. 2021. Exosomes released by imatinib‑resistant K562 cells contain specific membrane markers, IFITM3, CD146 and CD36 and increase the survival of imatinib‑sensitive cells in the presence of imatinib. Int J Oncol 58:238–250. doi:10.3892/ijo.2020.5163 33491750

[30] Li K, Markosyan RM, Zheng YM, Golfetto O, Bungart B, Li M, Ding S, He Y, Liang C, Lee JC, Gratton E, Cohen FS, Liu SL. 2013. IFITM proteins restrict viral membrane hemifusion. PLoS Pathog 9:e1003124. doi:10.1371/journal.ppat.1003124 PMC3554583 23358889

[31] Ono A, Freed EO. 2001. Plasma membrane rafts play a critical role in HIV-1 assembly and release. Proc Natl Acad Sci USA 98:13925–13930. doi:10.1073/pnas.241320298 PMC61143 11717449

[32] Shrivastava-Ranjan P, Bergeron É, Chakrabarti AK, Albariño CG, Flint M, Nichol ST, Spiropoulou CF. 2016. 25-hydroxycholesterol inhibition of lassa virus infection through aberrant GP1 glycosylation. mBio 7:e01808-16. doi:10.1128/mBio.01808-16 PMC5181775 27999160

[33] Lev S, Ben Halevy D, Peretti D, Dahan N. 2008. The VAP protein family: from cellular functions to motor neuron disease. Trends Cell Biol 18:282–290. doi:10.1016/j.tcb.2008.03.006 18468439

[34] Desai TM, Marin M, Chin CR, Savidis G, Brass AL, Melikyan GB. 2014. IFITM3 restricts influenza A virus entry by blocking the formation of fusion pores following virus-endosome hemifusion. PLoS Pathog 10:e1004048. doi:10.1371/journal.ppat.1004048 PMC3974867 24699674

[35] Wrensch F, Winkler M, Pöhlmann S. 2014. IFITM proteins inhibit entry driven by the MERS-coronavirus spike protein: evidence for cholesterol-independent mechanisms. Viruses 6:3683–3698. doi:10.3390/v6093683 PMC4189045 25256397

[36] Palmer AE, Jin C, Reed JC, Tsien RY. 2004. Bcl-2-mediated alterations in endoplasmic reticulum Ca2+ analyzed with an improved genetically encoded fluorescent sensor. Proc Natl Acad Sci USA 101:17404–17409. doi:10.1073/pnas.0408030101 PMC535104 15585581

[37] Jarrosson-Wuilleme L, Goujon C, Bernaud J, Rigal D, Darlix JL, Cimarelli A. 2006. Transduction of nondividing human macrophages with gammaretrovirus-derived vectors. J Virol 80:1152–1159. doi:10.1128/JVI.80.3.1152-1159.2006 PMC1346929 16414992

