# Supplementary material for: Functional redundancy, complementation, and cross-talk between CH25h and IFITM3 in infection and in IFITM3 trafficking and secretion
Source: J Virol. 2026 Sep 28;100(10):e01378-26. doi: 10.1128/jvi.01378-26 (PMC13712973; doi:10.1128/jvi.01378-26)
Supplement: Supplementary file 1 [file jvi.01378-26-s0002.docx]

**Supplemental Material for**

# Functional redundancy, complementation and cross-talk between CH25h and IFITM3 in infection and in IFITM3 Trafficking and Secretion

Yuxin Song^a^*, Tingting Xia^a^*, Charlène Martin^a^, Gaetan Despres^b^, Julien Burlaud-Gaillard ^c,d^, Antonin Lamaziere^be^, Philippe Roingeard ^c,d^ and Andrea Cimarelli^a^ #

Corresponding author: Andrea Cimarelli

Email: [acimarel@ens-lyon.fr](mailto:acimarel@ens-lyon.fr)

This PDF file includes:

Appendix Figures A1 to A3

**Appendix Figure A1. Effects of CH25h and IFITM3 expression on HIV-1 infectivity and effects of 25HC on cell growth.**

The data refers to Fig 1. **A)** Intracellular levels of IFITM3 and **(B)** cell numbers of SupT1 cells incubated with the indicated concentrations of 25HC and analyzed at the indicated days (n=3). **C)** The graph presents the non-normalized infectivity data of the corresponding graph of Figure 1D.

**Appendix Figure A2. Effects of CH25h and IFITM3 expression on VSV.**

The data refers to Fig 2. **A)** The graph presents the non-normalized infectivity data of the corresponding graph of Fig 2A. **B)** A549 cells expressing dox.-inducible forms of IFITM3 and/or CH25h were obtained upon retroviral-mediated gene transduction. Following the proteins induction with dox (1.5 μg/mL) for twenty-four hours, cells were either analyzed by WB or challenged with VSV-GFP prior to flow cytometry analysis 16-18 hours later. N=3; *, p<0.05 following Ordinary one-way Anova, Dunnett’s multiple comparison test.

**Appendix Figure A3. Effects of 25HC on cell viability and infectivity.**

**A)** Scheme of the approach used here. **B and C)** HEK293T cells were incubated with 25HC for twenty-four hours then either analyzed for viability (cell counting and trypan blue exclusion), or susceptibility to infection upon VSV-GFP challenge and flow cytometry analysis. The graphs present AVG and SEM of 3 independent experiments. *, p<0.05, according to a two-tailed, unpaired Student t test between the two indicated conditions.
